# *In vitro* studies on CNGRC-CPG2 fusion proteins for ligand-directed enzyme prodrug therapy for targeted cancer therapy

**DOI:** 10.18632/oncotarget.27478

**Published:** 2020-02-11

**Authors:** Layla Al-mansoori, Sara S. Bashraheel, Alanod D. Al Qahtani, C. David O’Connor, Philip Elsinga, Sayed K. Goda

**Affiliations:** ^1^Qatar University, Biomedical Research Center, Qatar University, Doha, Qatar; ^2^University of Groningen, University Medical Center Groningen, Groningen, The Netherlands; ^3^Protein Engineering Unit, Life and Science Research Department, Anti-Doping Lab-Qatar, Doha, Qatar; ^4^Department of Biological Sciences, Xi’an Jiaotong-Liverpool University, Science and Education Innovation District, Suzhou, China; ^5^Cairo University, Faculty of Science, Chemistry Department, Giza, Egypt

**Keywords:** targeted cancer therapy, carboxypeptidase G2, glucarpidase, ligand directed enzyme prodrug therapy, antibody directed enzyme prodrug therapy

## Abstract

The sequence asparagine-glycine arginine (NGR), flanked by Cysteine (Cys) residues so as to form a disulfide-bridge (CNGRC), has previously been found to target and bind specifically to aminopeptidase N (APN), which is highly expressed on the surface of tumor cells. The goal of this study was to develop and evaluate the potential of fusion proteins carrying the CNGRC sequence linked to the enzyme carboxypeptidase G2 (CPG2) for targeted cancer therapy. We refer to this strategy as ligand-directed enzyme prodrug therapy (LDEPT).

We constructed two forms of the CNGRC-CPG2 fusions, containing one or two copies of the cyclic NGR motif and designated CNGRC-CPG2 (X-CPG2) and CNGRC-CPG2-CNGRC (X-CPG2-X), respectively. *In vitro* binding assays of the purified constructs showed that both X-CPG2 and X-CPG2-X bound with high affinity to cancer cells expressing high levels of APN, compared to their binding to cells expressing low levels of APN.

Further *in vitro* studies of the constructs to assess the therapeutic potential of LDEPT were carried out using cells expressing high and low levels of APN. Using methotrexate, it was demonstrated that cancer cell survival was significantly higher in the presence of the fusion proteins, due to the hydrolysis of this cytotoxic drug by CPG2. Conversely, when the prodrug ZD2767P was used, cancer cell killing was higher in the presence of the fused CPG2 constructs than in their absence, which is consistent with CPG2-mediated release of the cytotoxic drug from the prodrug. Furthermore, the doubly-fused CPG2 construct (X-CPG2-X) was significantly more effective than the singly-fused construct (X-CPG2).

## INTRODUCTION

Metastasis is a key problem preventing cancer treatment, and often results in patient death. In the advanced stages of cancer, tumor cells break the extracellular matrix (ECM) barrier and invade additional tissues in a multistep process [1]. Two classes of enzymes known to be involved in the ECM degradation are aminopeptidase N (APN) and the matrix metalloproteases (MMPs). Targeted anti-metastatic agents have been developed against MMPs, but to date their efficacy with respect to cancer treatment appears to be limited [2].

APN, also known as CD13, is a transmembrane receptor with an exopeptidase activity, and is expressed in many tissues and cell types [3]. It plays a crucial role in metastasis by degrading the ECM thus allowing the escape of tumor cells into the bloodstream [4]. APN also promotes angiogenesis and the invasion of neo-endothelial cells through the ECM [5].

Several studies have reported that various types of cancer cells have abnormally high levels of expression of APN, which made it a promising target for cancer therapy [6]. Recent studies have developed synthetic APN inhibitors and tested their anti-metastasic potency. Some of these compounds showed a significant capacity to inhibit and/or reduce cancer metastasis *in vivo* and *in vitro* [7].

During the late 1990s, a small cyclic peptide (asparagine-glycine-arginine) (NGR), discovered via phage display libraries, was found to have tumor-homing properties and a high binding affinity to APN expressed by the neo-angiogenic endothelial cells. Among the various isoforms of CD13 expressed by cells, the CNGRC peptide binds specifically to a tumor-specific form of CD13 [8]. Moreover, deamination of CNGRC produces a peptide (isoaspartate-glycine-arginine) that recognizes αѴβ3 integrin, which is a highly expressed biomarker of angiogenic vessels [9]. Thus, the CNGRC peptide has been utilized as a carrier for many cancer-related applications, such as cancer cell imaging, and the development of novel anti-cancer compounds that could be targeted to tumor cells [10]. Early studies established that several anti-cancer drugs had increased potency when linked to CNGRC, due to their higher localized cytotoxicity. Since then, the peptide has been linked or fused with a variety of molecules including anti-cancer drugs (e. g. doxorubicin), cytokines (interferone γ “IFNγ” and human tumor necrosis factor “hTNF”), toxins (A), enzymes (cytosine deaminase “CD”, and truncated coagulase) in addition to fusion with other therapeutic proteins such as anti-epidermal frowth factor receptor “anti-EGFR” and an scFv antibody fragment [11–18].

Antibody-directed enzyme prodrug therapy (ADEPT) is an appealing method for treating tumors with a significantly reduced undesirable side effects. This is achieved by directing the enzyme-prodrug complex to the tumor site via a tumor-specific antibody. Several enzymes have been used in combination with a variety of drugs, but only an ADEPT system using carboxypeptidase G2 (CPG2) has reached clinical trials [19]. CPG2 is an exopeptidase that can be used clinically to convert synthetic non-toxic “benzoic mustard prodrugs” to cytotoxic moieties. Additionally, CPG2 is used in to detoxify patients who have inadvertently been given an overdose of methotrexate (MTX) [20, 21]. Methotrexate is used in chemotherapy for treatment of various cancers but has strong side effects on tissues, especially in the kidney, and can lead to renal dysfunction and failure. Accordingly, rapid removal of methotrexate is of great importance and this can be achieved by administration of CPG2, which acts by hydrolyzing the carboxyl terminal glutamate moiety of methotrexate to produce the safer products glutamic acid and 2,4- diamino-N10-methylpteroic acid (DAMPA) [22]. Because of the dual benefits of CPG2 in cancer treatments, it is considered an enzyme of great potential in this area of research.

**Figure 1:**
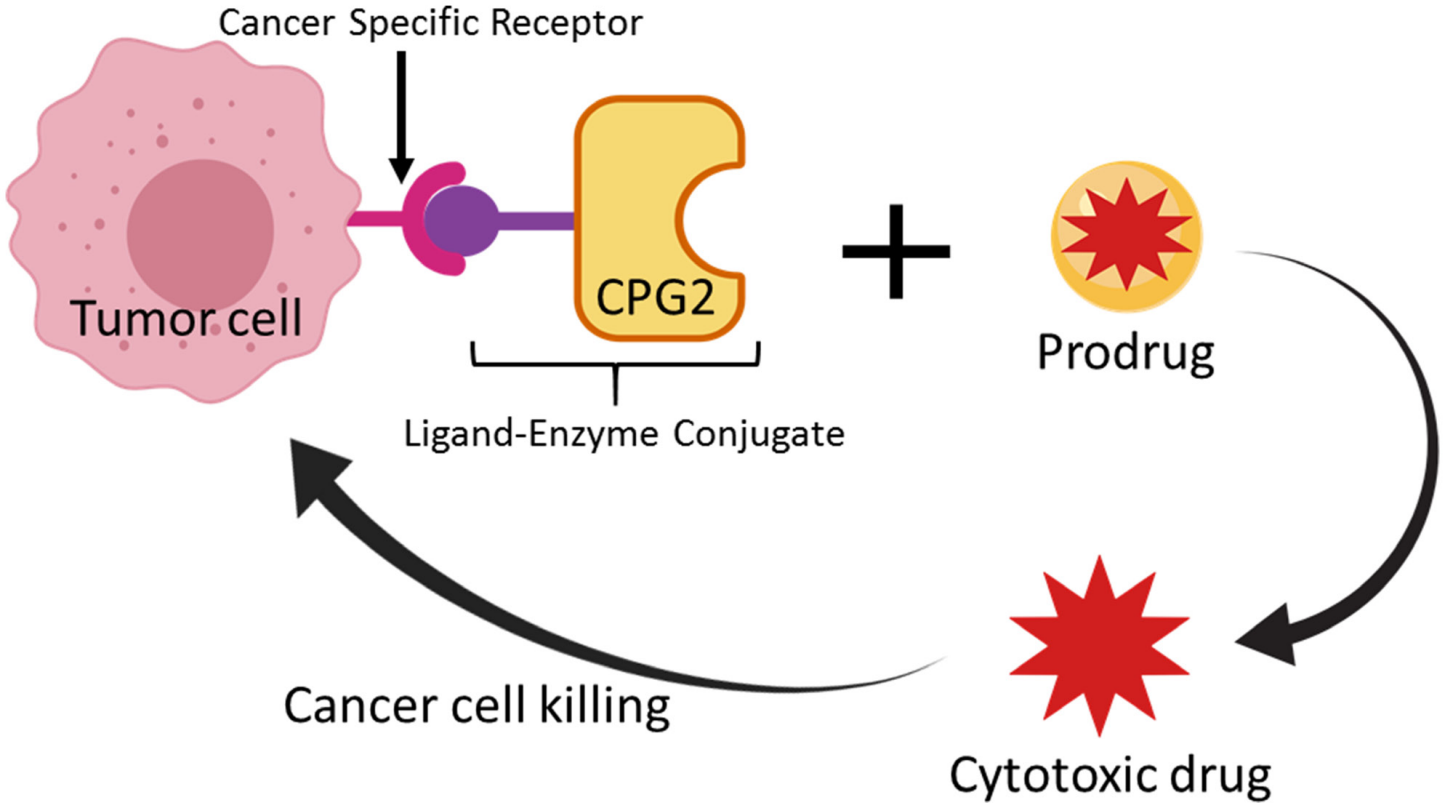
Schematic representation of the Ligand-Directed Enzyme Pro-Drug Therapy (LDEPT) strategy. Tumor cells that express cancer specific receptors (such as aminopeptidase N “APN”) are bound via a specific ligand (such as CNGRC) that is attached via a linker to a therapeutic enzyme (such as CPG2) that can convert an inactive pro-drug (ZD2767P) to a cytotoxic compound at the site of tumor cells.

In the LDEPT strategy, a protein or peptide ligand is used to direct the enzyme to the tumor site where the prodrug will be converted to cytotoxic drug resulting in cancer cell death (Figure 1). In contrast to ADEPT, where a relatively large antibody or antibody fragment is covalently attached to the enzyme, LDEPT results in smaller fusion proteins that are relatively cheap to produce and that are less likely to have solubility issues.

**Figure 2:**
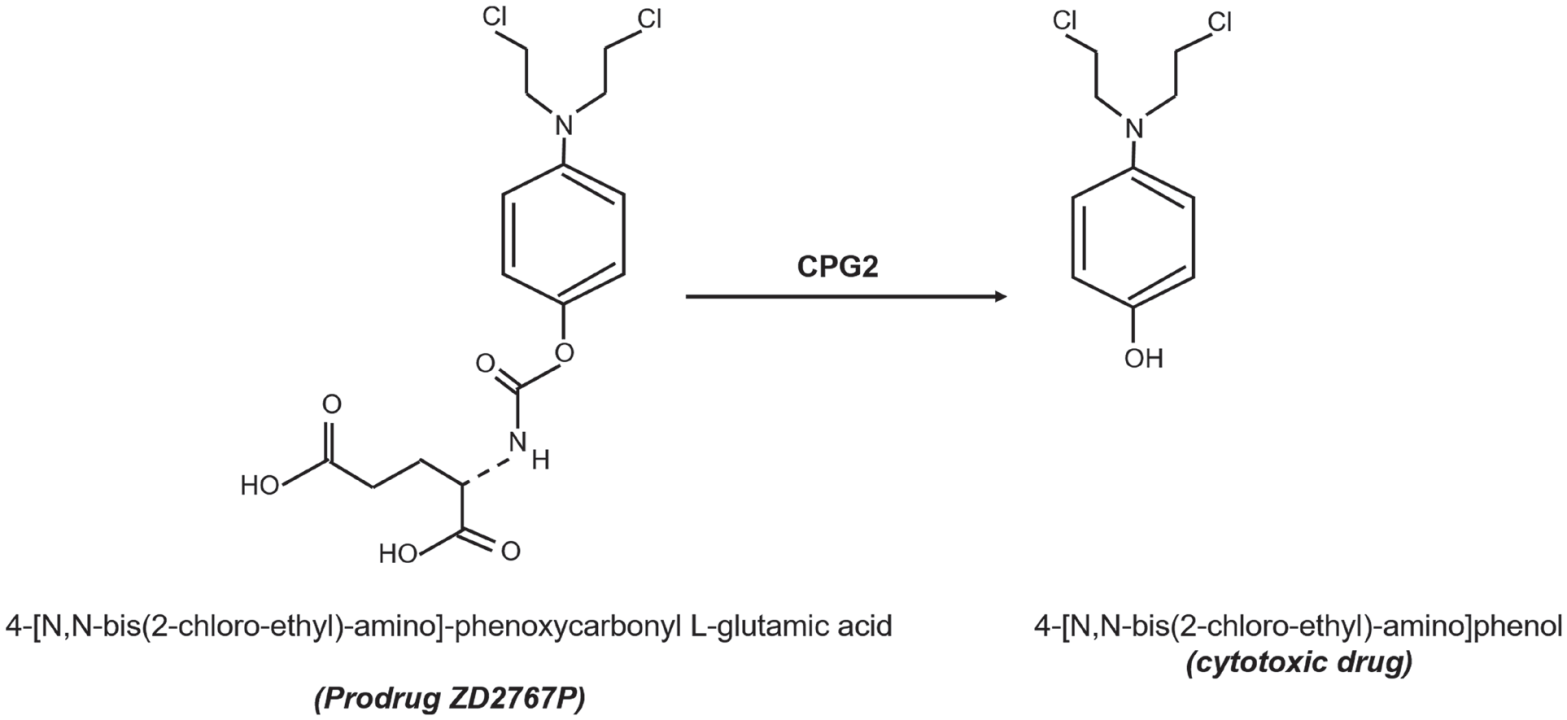
Structures of the prodrug compound (ZD2676P) used in this study and the cytotoxic compound produced following carbopeptidase G2 “CPG2”-mediated hydrolysis and removal of a glutamic acid moiety.

In this study, we used two derivatives containing a cyclic peptide (CNGRC) that binds with high affinity to APN and direct CPG2 to tumor cells. The targeting complexes were used in conjunction with the prodrug ZD2767P (an alkylating mustard agent), which is activated to a potent cytotoxic drug once CPG2 hydrolyses and cleaves the glutamate moiety [23] (Figure 2). We investigated the specific binding of the fusion proteins using cancer cell lines with different levels of APN expression. Furthermore, the cytotoxic effect of methotrexate and/or the prodrug ZD2767P was investigated following treatment with the fusion proteins.

## RESULTS

### Cloning, overexpression and purification of fusion proteins

**Figure 3:**
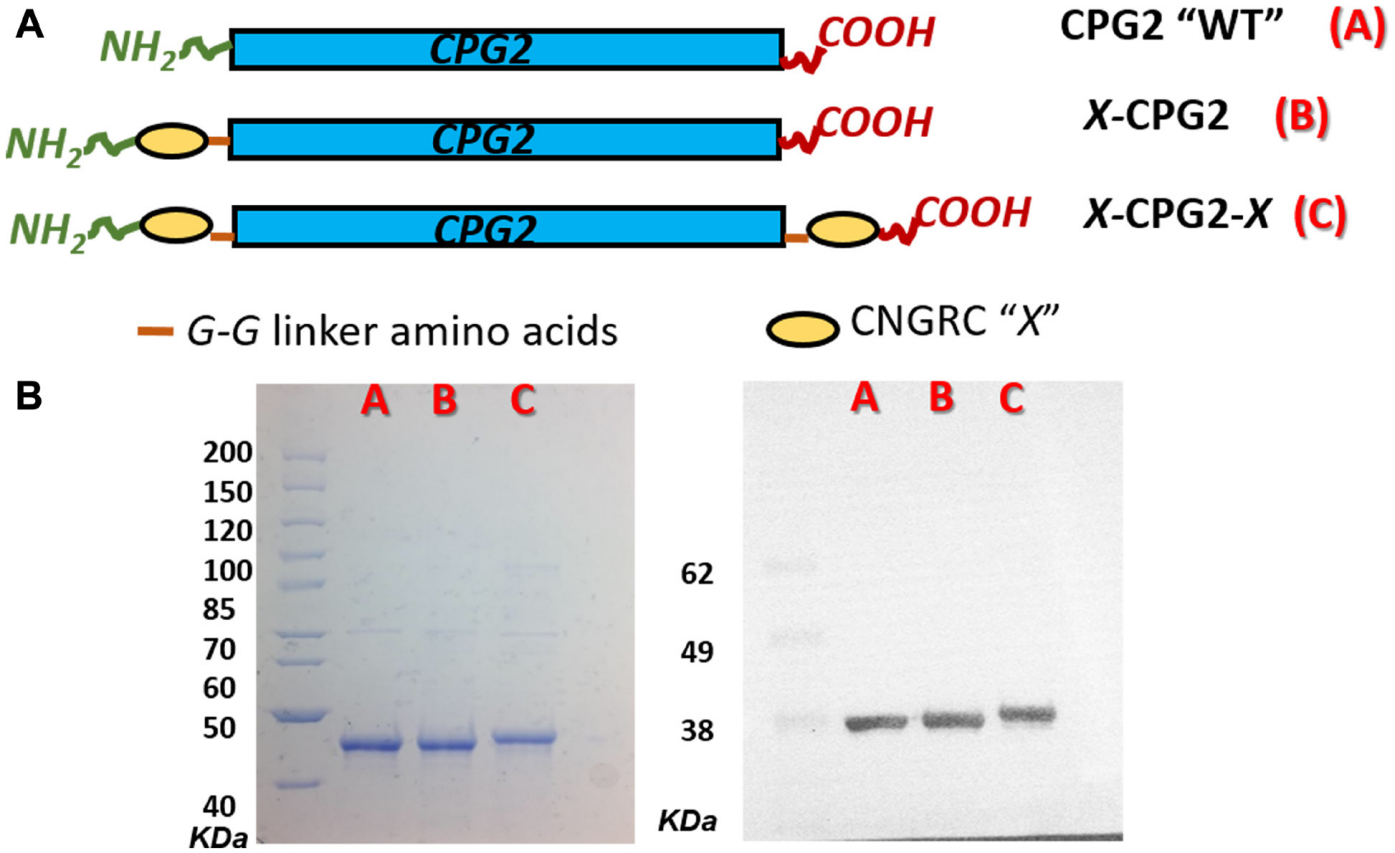
Cloning and production of CNGRC-CPG2 fusion proteins. (**A**) Schematic representations of the structures of the constructed fusion proteins. (**B**) Left-hand panel: SDS-PAGE analysis of the purified fusion proteins, lanes A, B and C show purified WT, single and double fusion proteins respectively. Right-hand panel: western blotting with anti-CPG2 antibody.

Two fusion proteins were constructed using CNGRC and His-tagged CPG2, as shown in Figure 3A. The first construct, designated CNGRC-CPG2 (X-CPG2), carried the cyclic NGR motif at the N-terminus while the second, designated CNGRC-CPG2-CNGRC (X-CPG2-X), had an additional cyclic NGR motif at the C-terminus. Sequencing of the plasmids carrying the fusion proteins confirmed the predicted structures of the constructs (Table 1). The encoded proteins were expressed and purified, resulting in a final yield of 3.6 mg, 5 mg and 6.2 mg of purified WT, single and double fusion proteins, respectively, per g cell paste. SDS-PAGE analysis of the purified proteins and immunoblotting using an anti-CPG2 antibody both showed that the doubly-fused protein was discernibly larger in size (Figure 3B).

**Table 1:** Nucleotide and peptide sequences of the CNGRC-CPG2 fusion proteins constructed clones^1^

*Nucleotide sequence*	CPG2 “WT”	CATATGGCCCTGGCCC................. CAAGGGTTGAAAGCTT
	*X*-CPG2	CATATG**TGCAACGGTCGTTGT** *GGTGGT*GCCCTGGCCC.............. CAAGGGTTGAAAGCTT
	*X*-CPG2-*X*	CATATG**TGCAACGGTCGTTGT** *GGTGGT*GCCCTGGCCC............. CAAGGGTTG*GGTGGT* **TGCAACGGTCGTTGT**AAAGCTT
*Peptide sequence*	CPG2 “WT”	H Met A L A Q........ D L G A G K G Stop K L
	*X*-CPG2	H Met **C N G R C** *G G* A L A Q.......... D L G A G K G Stop K L
	*X*-CPG2-*X*	H Met **C N G R C** *G G* A L A Q............. D L G A G K G *G G* **C N G R C** Stop K L

^1^The linker sequence is indicated in italic while the sequence in bold is for the fused protein part.

### Catalytic activity assay of fusion proteins

**Figure 4:**
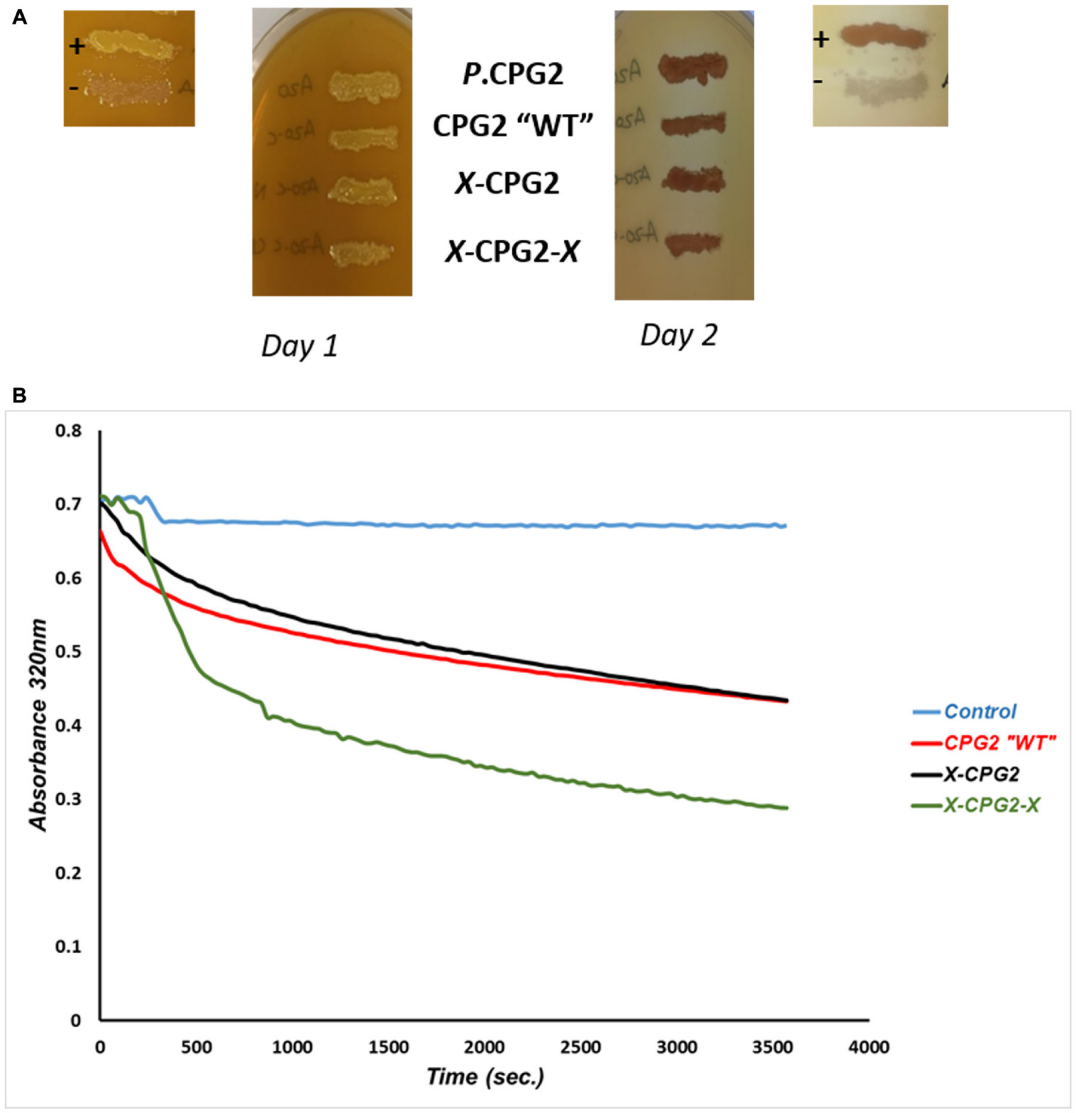
Catalytic activity and kinetics of CPG2 and its fusion protein derivatives. (**A**) *E. coli* BL21 (DE3) RIL cells expressing different CNGRC-CPG2 fusion proteins grown on folate supplemented agar plates. The yellow precipitates indicate CPG2 proteolytic activity. (+) and (-) are positive and negative controls respectively. *P*. CPG2: *Pseudomonas putida* CPG2 was used as a further positive control. (**B**) A graph showing the rate of methotrexate “MTX” hydrolysis by CNGRC-CPG2 proteins. Equal amounts of the proteins were used in these assays.

*E. coli* strains expressing each fusion protein were grown on agar plates supplemented with folate, as a qualitative check that the fusion proteins retained CPG2 activity. After 24 hours of incubation, all clones produced a yellow precipitate, similar to the positive control, and developed a clear zone surrounding the area of bacterial growth by Day 2 (Figure 4). CPG2 activity associated with the fusion proteins was then assessed quantitatively by measuring the hydrolysis of methotrexate. The kinetic parameters, maximal initial velocity (V_max_), Michaels-Menten constant (K_m_) and turnover number (K_cat_) were calculated. The results showed a significant increase in the V_max_ of the enzyme when two copies of the CNGRC motif fused to CPG2, relative to the construct with a single copy of the motif and non-fused WT CPG2 (Table 2).

**Table 2:** Enzyme kinetics parameters of CPG2 and its CNGRC fusion derivatives

	CPG2	*X*-CPG2^1^	*X*-CPG2-*X*^1^
*K*_cat_ “min^-1^”	35.64 ± 1.4	29.8 ± 2.06	72.3 ± 1.7
V_max_ “µM/min/µg”	75.5 ± 3.5	63.23 ± 5.1	153.3 ± 2.9
K_m_ “µM”	235.6 ± 12.1	171.7 ± 16.5	676 ± 24.07

^1^X indicates the position (s) of the CNGRC peptide fused to CPG2.

### CD spectra

**Figure 5:**
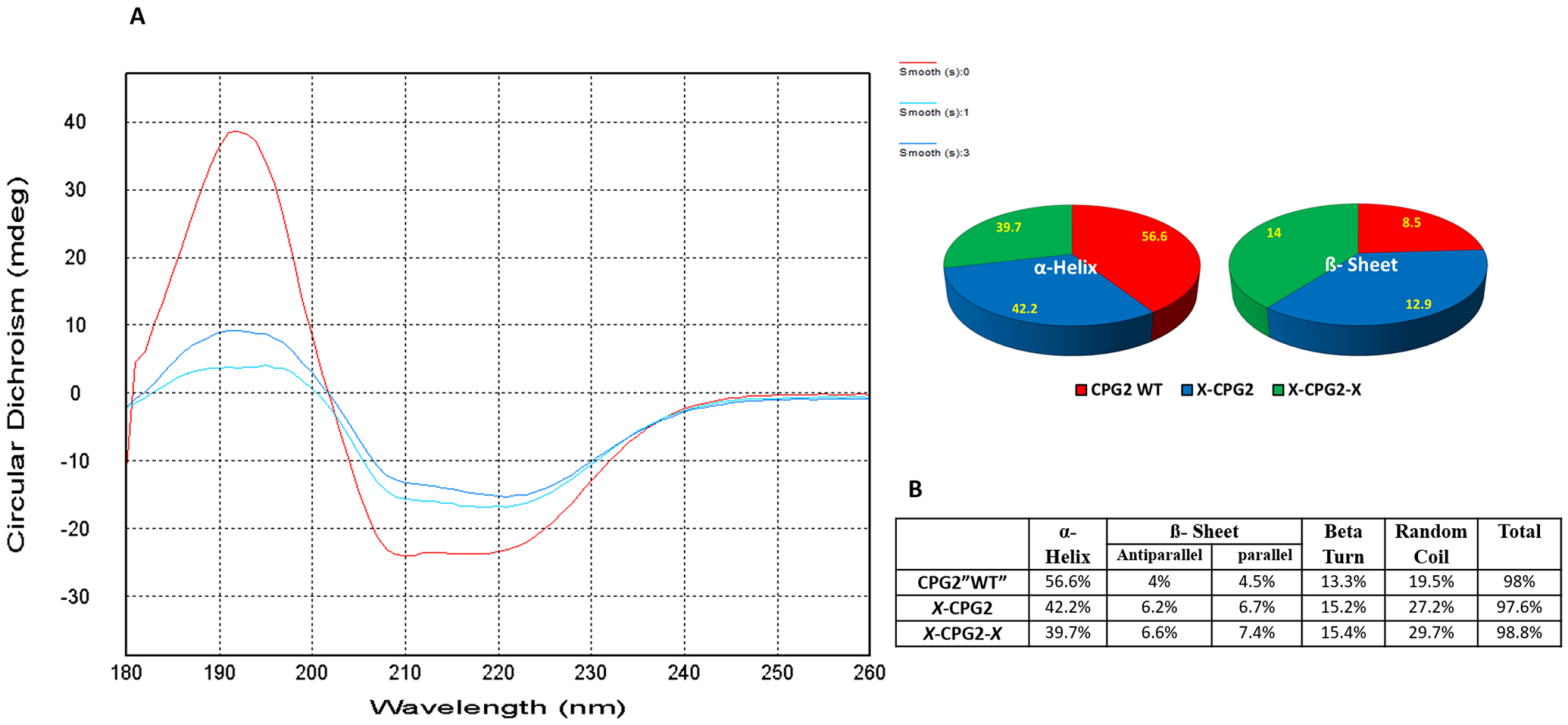
Far UV circular dichroism profiles of CPG2 and its fusion derivatives with the distribution of the β sheets and α-helix. The figure shows combined molar ellipticity data for CPG2 (red line), the single fused construct (darker blue line) and the double fused construct (cyan line). ‘smooth 0’: CPG2, ‘smooth 1: X-CPG2, and ‘smooth 3′: X-CPG2-X. The obtained spectral data were corrected for the baseline buffer.

Circular dichroism was used to assess the degree of conformational change in the fusion proteins relative to WT CPG2. The specific shape of the measured far UV CD signals for fusion proteins (single and double) relative to the WT CPG2 is shown in Figure 5A. The calculated secondary structure (Figure 5B) indicates changes in the structural composition of CPG2 protein when fused with CNGRC (single “X-CPG2” and double fusion “X-CPG2-X” proteins). The percentage of alpha helix is reduced whereas there is an increase in the percentage of beta sheet structure.

### *Ex-vivo* immunogenicity of the fusion proteins and their stability in serum

**Figure 6:**
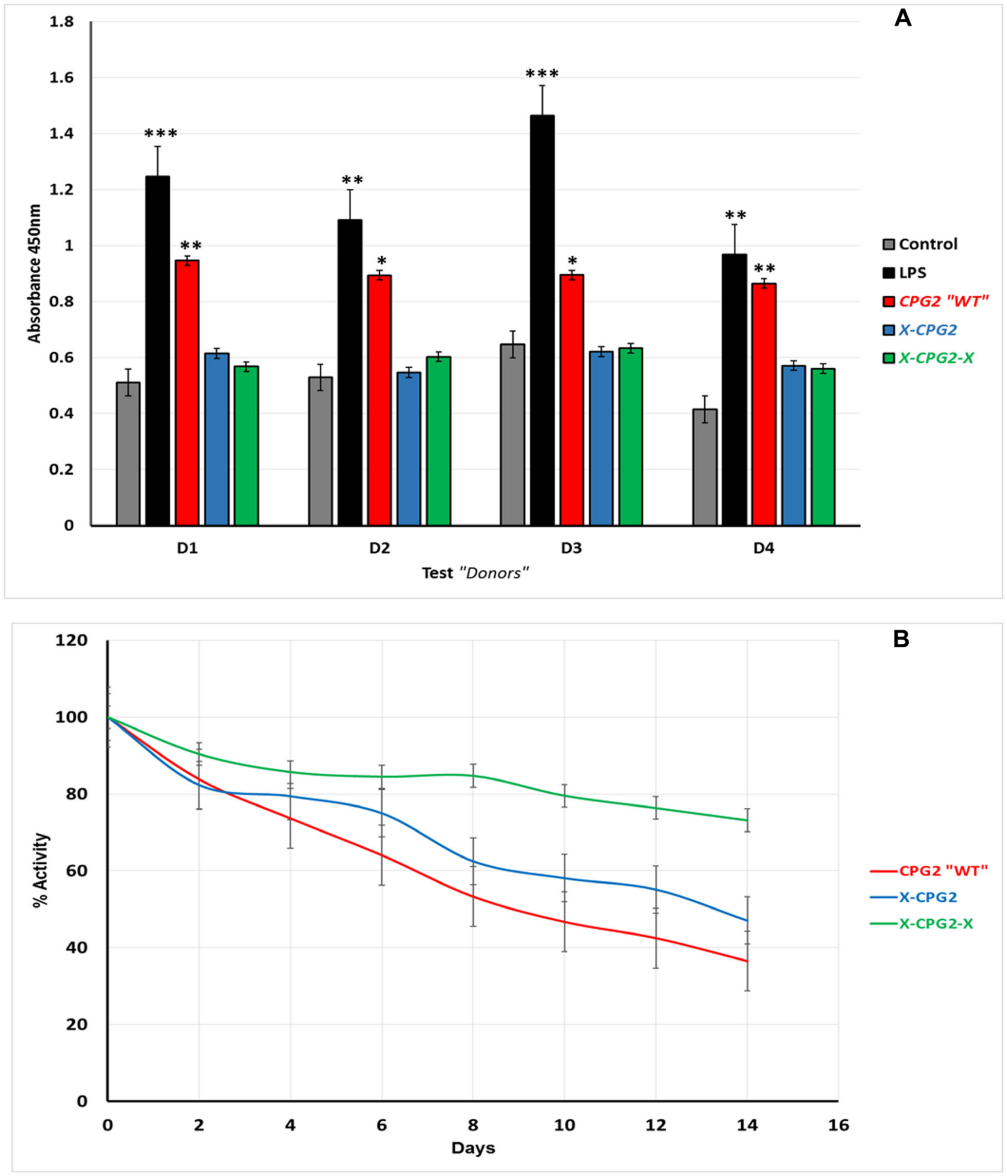
(**A**) Immunogenicity of CPG2 and its fusion protein derivatives. Human PBMCs from four donors were used as described in the Materials & Methods section. Cells treated with fusion proteins (*X-*CPG2 and *X*-CPG2-*X*) showed no significant differences in immunogenicity compared with the control groups. Student *t*-test used for the resulting values and relative comparison with the control non-treated cells “^*^ *P* < 0.05, ^**^ *P* < 0.001” (**B**) Stability of CPG2 and its fusion protein derivatives in human serum at 37°C. The catalytic activity of the proteins was assayed every 48 h over a period of 14 days.

To test the immunogenicity of the generated fusion proteins, a T cell proliferation assay was used (Fu et al., 2014). Unfractionated PBMCs from four healthy donors were incubated with the purified endotoxin-free fusion proteins (WT, single and double) for 48 hours. According to the CCK-8 proliferation assay results, there was no significant induction in cell proliferation following incubation with fusion proteins, compared with the vehicle control groups. In contrast, T-cells were significantly induced with LPS (positive) groups and also by the non-fused CPG2 (Figure 6A). These results suggest that the fusion proteins have reduced immunogenicity relative to native CPG2. Analysis of the stability of the fusion proteins in human serum indicated that they were more stable than the non-fused WT. Furthermore, the double fusion protein, X-CPG2-X, showed higher stability, with more than 70% activity remaining after 12 days of incubation in human serum at 37°C (Figure 6B).

### Differential binding affinities of fusion proteins to cancer cell lines expressing APN

**Figure 7:**
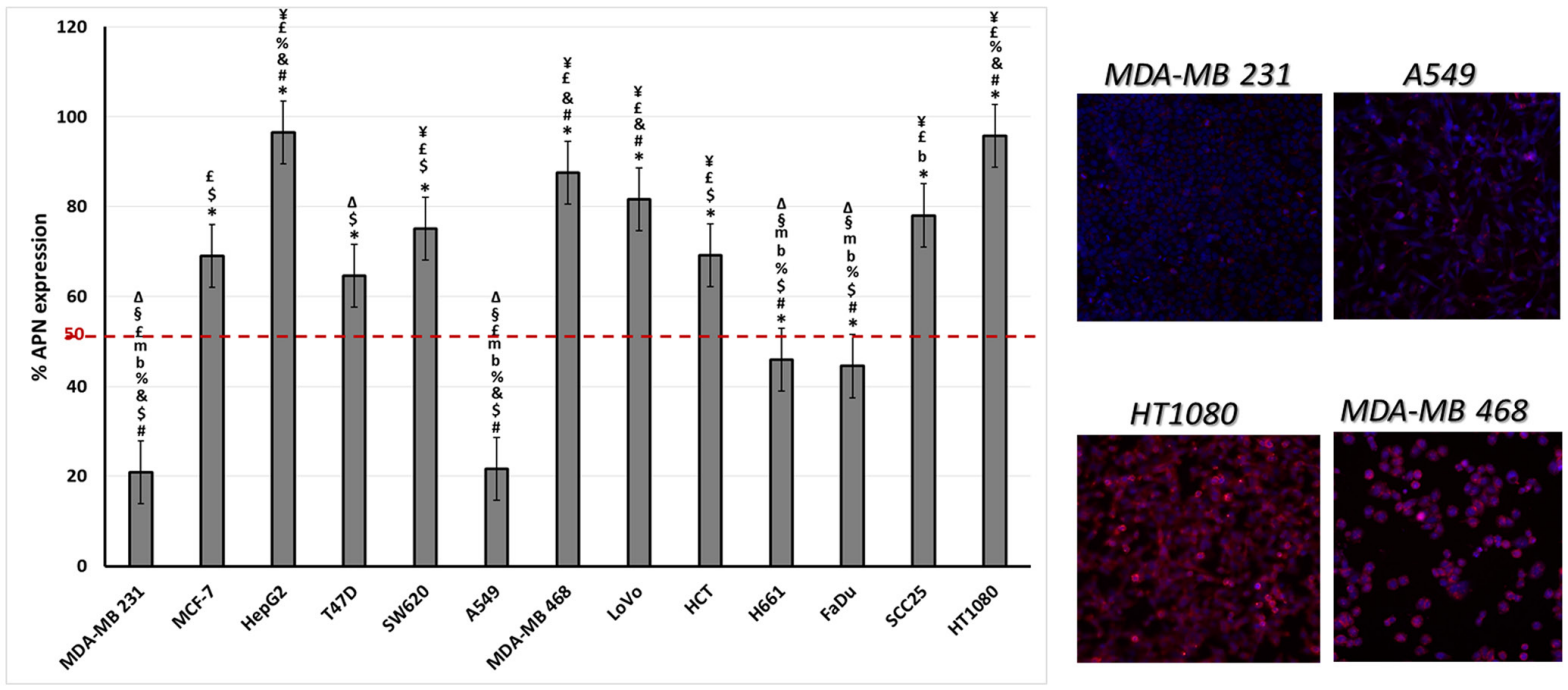
The levels of APN expression in various cancer cell lines. Left-hand panel: Different cancer cell lines were labeled with anti-CD13 antibody and the percentage of CD13 expression was determined as described in the Materials & Methods section. One way ANOVA with post hoc Tukey’s test for the resulting values presented with the following symbols indicating statisitical significance difference “*p* < 0.001” in comparison to the stated cell lines: * to MDA-MB231, # to MCF-7, $ to HepG2, & to T47D, % to SW620, b to MDA-MB 468, m to LoVo, £ to H661, ¥ to FaDu, § to SCC25 and ∆ to HT1080. Right-hand panels: fluorescence imaging of two low (MDA-MB 231 and A549) and two high (HT1080 and MDA-MB 468) APN expressing cell lines. An antibody specific for CD13 was detected using a secondary antibody labelled with Alexa fluor 488 (red) while nuclei were labelled with DAPI (blue).

**Figure 8:**
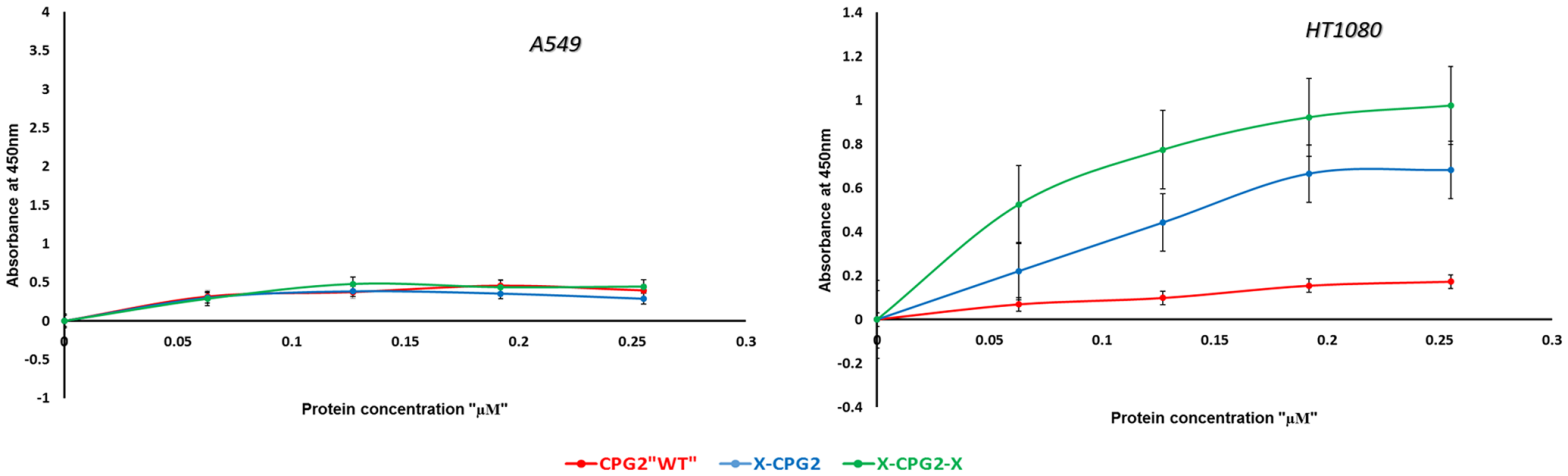
*In vitro* binding assay of CPG2 and its fusion protein derivatives to cancer cells expressing low (A549) and high (HT1080) levels of APN. Binding was measured as described in the Materials & Methods section.

The level of APN expression in different cancer cell lines was determined using anti-CD13 antibody labeling. The results showed a range of levels of APN expression (Figure 7). Some were high (e. g. MDA-MB 468 and HT1080 cell lines) while others were low (e. g. the MDA-MB 231 and A549 cell lines). Two cancer cell lines were used to test the binding affinity of the fusion proteins, low APN expressing (A549) and high APN expressing (HT1080) cell lines. Both the single and double fused proteins bound to the high APN expressing cells (HT1080), with increasing concentrations. In contrast, WT CGP2 did not show significant binding to HT1080 cells (Figure 8). Interestingly, the double fused protein, X-CPG2-X, displayed higher binding affinity (K_d_ 0.4 ± 0.11), compared with the single fused protein (K_d_ 0.91 ± 0.15). Neither the fusion proteins nor WT CPG2 showed significant binding to the low APN expressing cell line (A549).

### *In vitro* cytotoxicity of MTX or ZD2767P on cancer cell lines treated with the fusion proteins

**Figure 9:**
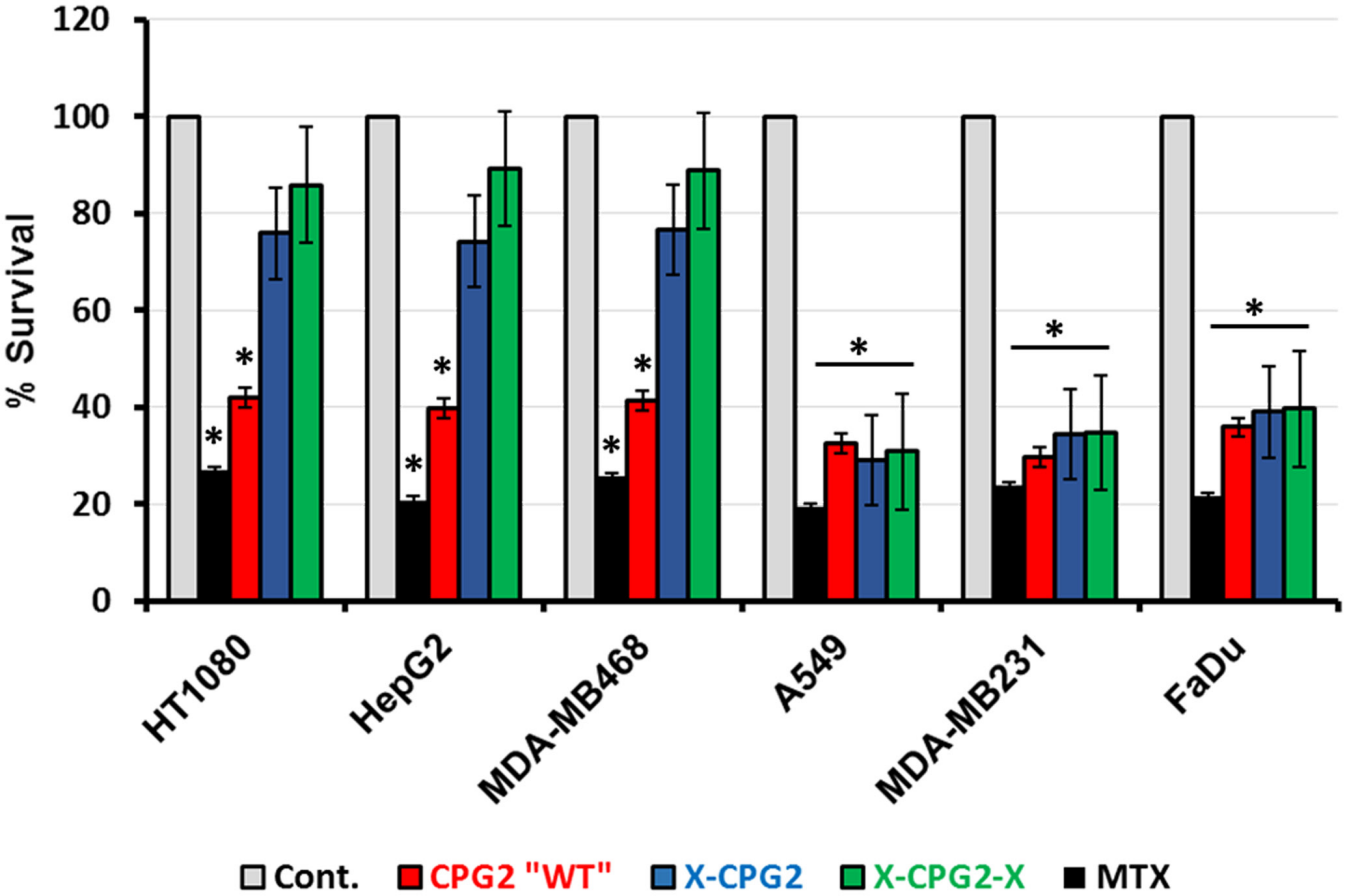
Methotrexate detoxification by CPG2 and its fusion protein derivatives using cells expressing high- or low-levels of APN. Cancer cell lines expressing low levels of APN (A549, MDA-MB231 and FaDu) or high levels of APN (HT1080, HepG2 and MDA-MB468) were treated with MTX. Cell viability was then measured as described in the Materials & Methods section. Two groups served as negative (non-MTX treated) and positive (treated with MTX only) controls. The results show the percentage of viable cells obtained following MTT assay. “student *t*-test ^*^ *P* < 0.001 relative to the non-treated control”.

Previous studies have shown that fusion proteins containing CPG2 have potential for the removal of excess methotrexate (MTX) during chemotherapy. We therefore investigated the degree of MTX cytotoxicity on cells that had been treated with the fusion proteins. Cell survival was significantly higher in the high APN expressing cells that had been pre-treated with either single (X-CPG2) or double (X-CPG2-X) fused proteins, compared with the positive controls, i. e. cells treated only with the MTX (Figure 9). In contrast, there was no significant difference in the survival of low APN expressing cells pre-treated with the fusion proteins (Figure 9).

We next investigated whether the fusion proteins could be used to increase the degree of killing mediated by the prodrug ZD2767P. In a previous study, it was shown that the prodrug ZD2767P produced a highly potent cytotoxic drug with a short in plasma half-life of 14.5 min [24].

As shown in Figure 9, cells expressing high levels if APN (HT1080, MDA-MB486 and HepG2) showed significantly lower levels of survival with ZD2767P (about 50% lower) when treated with the fusion proteins relative to the same cells treated with either the non-fused CPG2 protein or the controls. In contrast, the percentage survival of cells expressing low levels of APN (A549, FaDu and MDA-MB231) was not significantly affected by the prodrug ZD2767P.

## DISCUSSION

Conventional cancer treatments such as chemotherapy and radiotherapy lack much specificity for cancer cells and hence also kill many healthy cells. In contrast, antibodies have the potential to deliver with accuracy therapeutic enzymes and drugs to tumors, due to their specificity for cognate antigens.

One targeted cancer strategy that exploits antibody specificity is antibody-directed enzyme prodrug therapy (ADEPT) [25, 26], which produces a cytotoxic drug in the vicinity of cancer cells using an enzyme (e. g. CPG2) linked to cancer-specific antibody and a prodrug. One potential advantage of ADEPT is that any cancer cell that does not express the required antigen may still be killed due to the bystander effect of the released cytotoxic agents. The ADEPT system has been successfully used in several clinical trials resulting in encouraging tumor responses [27]. However, two major problems may decrease its efficacy: (i) the limited choice of tumor markers that can be targeted by antibodies and (ii) enzyme immunogenicity, where several clinical trials have resulted in high system toxicity due to repeated administration of non-human enzymes [28].

To circumvent these issues, the present study investigated the use of peptides, fused with CPG2, that bind specifically to aminopeptidase N (APN), which is often highly expressed on the surfaces of many types of cancer cell. APN is a cancer cell marker known to have a major role in cancer cell invasion and angiogenesis [29]. It is highly expressed on the cell surface in various solid tumors but generally has low levels of expression in normal cell types. Different isoforms of APN have been found to be expressed by tumor cells, which has allowed the development of reagents that bind with high specificity to cancer cells. Using phage display libraries, peptides with the sequence Asparagine – Glycine – Arginine (NGR) sequence, flanked by Cys residues so as to form a disulfide bond (CNGRC), were found to bind to a tumor cell-specific isoform of APN isoform as well as to tumor blood vessels [30]. Accordingly, to pursue our goal of improving tumor-specific cytotoxicity, we designed two fusion proteins with the potential to target this APN isoform. The first fusion protein, CNGRC-CPG2 (X-CPG2), consists of the cyclic peptide at the N-terminus, a glycine-glycine linker and then CPG2 whereas the second has an additional CNGRC moiety at the C-terminus (X-CPG2-X) (Figure 3A). Our studies indicate that addition of the second copy of the CNGRC sequence enhances protein solubility, resulting in higher yields of the purified fusion protein (data not shown).

To ensure that the fusion of the peptides did not affect CPG2 activity we carried out a series of enzyme kinetic studies. The data indicate that addition of a single copy of CNGRC to CPG2 had no significant effect on the enzyme’s activity. We found, however, that the double fusion construct (i. e. with a CNGRC peptide at both the N- and C-termini of CPG2) had significantly increased enzyme activity comparing to the wild type and the single fused CPG2 (Figure 4). We speculate that the presence of the additional peptide at the C-terminus causes a conformational change that leads to an increase in substrate binding.

In keeping with these results, CD spectroscopic analysis in the far UV region showed a substantial difference in the secondary structure of CPG2 protein following double fusion with CNGRC, compared with non-fused CPG2. Specifically, there was decrease in the percentage of alpha helical structure, which was corroborated by the CDNN deconvolution. This conformational change is likely to explain the higher K_cat_ value for double fusion protein (X-CPG2-X) (Table 2). The higher enzyme activity of the double fused protein suggests that it could be useful not only in LDEPT but also in the detoxification of methotrexate in overdose cases.

One drawback of ADEPT is the immunogenicity of the CPG2 enzyme, which becomes problematical due to the need for repeated cycles of treatment. We therefore carried an *ex-vivo* assessment of the immunogenicity of the fusion proteins described here. Using a T-cell proliferation assay, we found that the fusion proteins appeared to cause no significant immunogenic response. However, it remains to be seen if the proteins become immunogenic following repeated administration to patients.

Encouragingly, we found that most of the enzymatic activity of CPG2 was retained following incubation with serum for 14 days (Figure 6A). In particular, the double fusion protein had ˃70% of its original catalytic activity after the incubation for two weeks in human serum (Figure 6B). In short, the data obtained so far indicate that the double fused CPG2 has enhanced enzyme activity, is less immunogenic and more stable than the free CPG2.

In view of these results, we investigated the binding of the fusion proteins to cancer cell lines expressing high- or low-levels of APN. Our results indicate that the fusion proteins bind strongly to cells expressing high levels of APN (i. e. the HT1080 cell line). Furthermore, the double fusion protein had a significant increase in binding affinity compared to the fusion protein with a single copy of the CNGRC peptide (Figure 8). Such higher binding affinity is expected to provide higher specificity and more restricted cytotoxic drug action at tumor sites.

Two *in vitro* tests of the LDEPT strategy were carried out. in the first, we examined the ability of the fusion constructs to convert the cytotoxic compound methotrexate to a much less toxic product [20]. As expected, APN expressing cells that had been pre-incubated with the proteins showed significantly less killing when exposed to methotrexate (Figure 9). In the second test we used the prodrug ZD2767P which is one of the alkylating mustard prodrugs known to be converted by the enzyme CPG2 into a highly cytotoxic drug that induces formation of DNA interstrand cross-links. This leads to arrest of cell growth (cytostatic effect) with induction of apoptosis [31].

**Figure 10:**
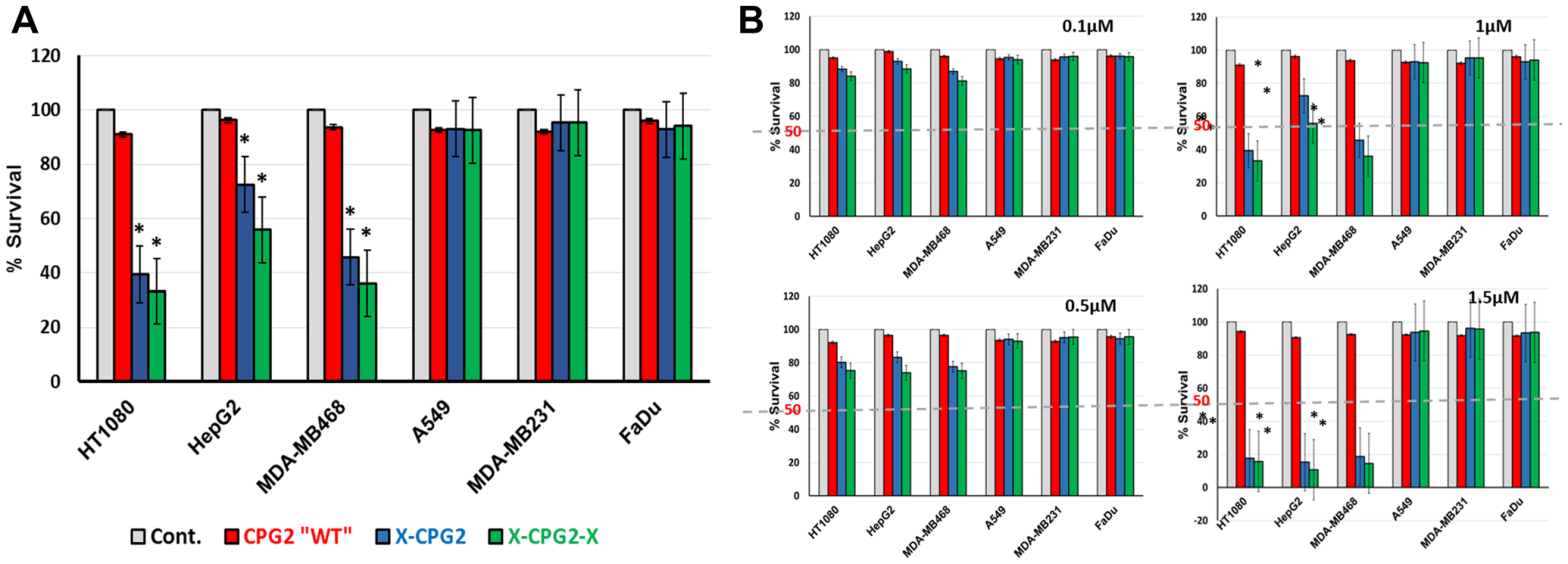
Cell killing mediated by CPG2 and its fusion protein derivatives following addition of the prodrug ZD2767P. (**A**) Cancer cell lines, expressing low or high levels of APN, were pre-incubated with CPG2 or its fusion protein derivatives, prior to addition of ZD2767P. Cell viability was then measured as described in the Materials & Methods section. “student *t*-test ^*^ *P* < 0.001 relative to the non-treated control”. (**B**) same as A but at different concentrations of the prodrug.

**Figure 11:**
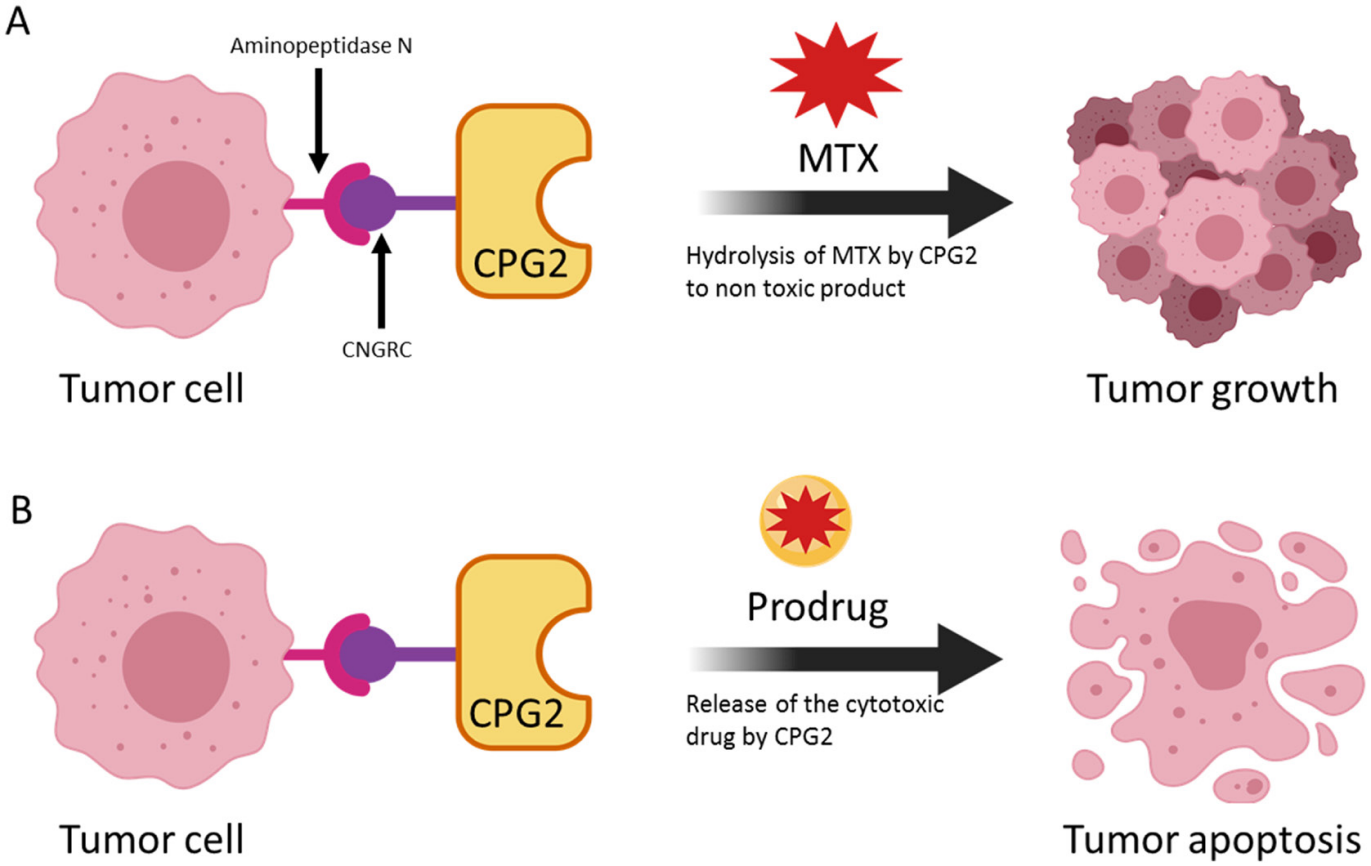
The use of a cytotoxic drug and a prodrug to demonstrate two uses of the LDEPT strategy. In (**A**), accumulation of CPG2 on the surface of cancer cells leads to removal of methotrexate (MTX) thereby protecting cells from killing. In principle, this effect could be used to remove an excess of MTX. In (**B**) enzyme-mediated conversion of a prodrug to a cytotoxic compound leads to tumor killing.

We exposed APN expressing cancer cells, pre-treated with the fusion proteins or with controls, to the prodrug, ZD2767P. The results clearly show that there was a significant increase in cell death in the presence of the fusion proteins (Figure 10). Importantly, in both tests, the fusion proteins only exhibited their effects when the cell lines used expressed high levels of APN. Figure 10A shows the effect of the prodrug at different concentration. Figure 11 shows the two strategies we used to investigate our new conjugates in LDEPT.

## MATERIALS AND METHODS

### Bacterial strains, vectors, and growth conditions

*Escherichia coli* (*E. coli*) DH5α™ (Thermo Fischer Scientific) was used as a cloning host, whereas *E. coli* BL21(DE3) RIL (Novagen) was used as an expression host. Plasmids pEt28a (Novagen, Stratagene) was used for cloning of the single (CNGRC-CPG2) and double (CNGRC-CPG2-CNGRC) fusion proteins. These bacterial strains were grown in Luria Bertani broth (LB Rich Media), which was obtained from Formedium. Agar (Formedium) was added to solidify the LB media.

### Restriction endonucleases, antibodies and other reagents

Restriction enzymes and DNA modifying enzymes were obtained from New England Biolabs. Vector dephosphorylation was performed using calf intestinal alkaline phosphatase (CIAP) that was purchased from Thermo Fischer Scientific. GeneJET Plasmid Miniprep and GeneJET Gel Extraction kits were obtained from Thermo Fischer Scientific, while recombinant proteins were purified by affinity chromatography using Ni coated resin (Sigma). The DNA and protein markers used (Quick-Load^®^ Purple 1 kb DNA Ladder and SeeBlue Plus2 Prestained ladder (198-10 kDa) were from NEB and Thermo Fischer Scientific, respectively. For immunoblotting, nitrocellulose membranes were from Thermo Fischer Scientific while an anti-6 His tag antibody mouse 6×-His Tag Monoclonal Antibody (HIS. H8) ws obtained from Invitrogen and used at 1:2500 dilution as a primary antibody. Rabbit polyclonal anti Xen-CPG2 antibody (GE Healthcare) was used at 1:3000 dilution while rabbit anti-polyethylene glycol antibody [PEG-B-47] (Abcam; ab51257) was used at 1:20,000 dilution. The secondary antibodies goat anti-mouse IgG H&L (HRP) (ab205719) and goat anti-Rabbit IgG H&L (HRP) (ab6721) were obtained from Abcam and used at 1:4000 and 1:5000, respectively. For detection of western blotting, an ECL chemiluminescent detection reagent (GE Healthcare) was used as substrate. For protein dialysis and buffer exchange, dialysis tubes (Spectra/Por 7 Dialysis Tubing, 10 kDa MWCO, 24 mm Flat-width, 5 meters/roll (16 ft) obtained from Spectrum was used. Immunogenicity assays used the following kits in accordance with the manufacturers’ instructions: Pierce™ High Capacity Endotoxin Removal Resin (Thermo Fisher Scientific); Pierce™ LAL Chromogenic Endotoxin Quantitation Kit (Thermo Fisher Scientific) and Cell Counting Kit – 8 (Sigma-Aldrich). The cell lines used were: MDA-MB468 (human breast adenocarcinoma), SW620 (human colorectal adenocarcinoma), MDA-MB231 (human breast adenocarcinoma), MCF7 (human breast adenocarcinoma), A549 (human lung carcinoma), HT-1080 (human fibrosarcoma), HepG2 (human hepatocellular carcinoma), T47D (human mammary ductal carcinoma), LoVo (human colorectal adenocarcinoma), HCT (human colorectal carcinoma), H661 (human large cell lung cancer), FaDu (human pharyngeal squamous cell carcinoma), SCC25 (human tongue squamous cell carcinoma). Cell lines were purchased from the American Type Culture Collection and cultured in the recommended media recommended by the ATCC. ZD2767P was supplied by Inovapharm Ltd. as a crystalline product. 1 mg of the solid prodrug was dissolved in 1 ml of sterile DMSO to make a 2 mM stock solution.

### Designing and construction of the single and double fusion protein clones

Cloning of the gene encoding a His-tagged version of the CPG2 enzyme from *Xenophilus azovorans* SN213 has been previously described [32]. The following PCR primers were used to prepare DNA fragments encoding the single (CNGRC-CPG2) and double (CNGRC-CPG2-CNGRC) protein constructs:

5′-GGAACCCATATGTGCAACGGTCGTTGTGGTGGTGCCCTGGCCCAGAAGCGCGAC-3′ and 5′-AAGCTTTCAACCCTTGCCGGCGCCCAGATCCATGATCAGGCGGGCCGCCATGTACAGGCGGCG-3′ for the single fusion; 5′-GGAACCCATATGTGCAACGGTCGTTGTGGTGGTGCCCTGGCCCAGAAGCGCGAC-3′ and 5′-AAGCTTTCAACAACGACCGTTGCAACCACCACCCTTGCCGGCGCCCAGATCCATGATCAGGCGGGCCGC-3′ for the double fusion. The resulting PCR products were confirmed by sequencing, cut with restriction enzymes *Hind*III and *Nde*I, and ligated into the similarly digested and dephosphorylated vector pET28a. To confirm the structure of the clones, plasmid minipreps were prepared and sequenced using the T7 promoter and terminator primers, T7F: 5′-TAATACGACTCACTATAGGG-3′ and T7 terminator 5′-GCTAGTTATTGCTCAGCGG-3′ (Eurofins).

### Expression and purification of fusion proteins

Plasmids encoding CPG2, CNGRC-CPG2 and CNGRC-CPG2-CNGRC were transfected into competent *E. coli* BL21 (DE3) RIL cells for protein expression. The cells were then grown at 37°C in LB media supplemented with kanamycin and chloramphenicol. When the cultures reached the required absorbance at 600 nm (0.5–0.6), recombinant protein production was induced with IPTG before further incubation at 4°C overnight. The following day, the cells were collected, resuspended in the lysis buffer (20 mM Tris, 100 mM NaCl and PMSF, pH7.4) and sonicated to open the cells. The resulting suspension was centrifuged, and the soluble protein collected in the supernatant. For protein purification, lysates were passed through HIS-Select Nickel Affinity Gel for protein capture via the His tag associated with the CPG2 protein. After washing and elution with imidazole, the protein was dialysed. The purified proteins were separated by SDS-PAGE and stained with Brilliant Coomassie blue.

### Catalytic activity assay of fusion proteins and serum stability

The enzymatic activity of the resulting purified proteins was determined by measuring the rate of methotrexate (MTX) hydrolysis [33]. 590 µl of 0.1 M Tris-HCl pH 7.3 containing 0.2 mM ZnSO4 and 5 µl of MTX (0.45 mM) was equilibrated at 37°C for 10 minutes then the total protein extract of each fusion protein (50 µg/ml) was added and incubated at 37°C. Samples were taken at 10 min intervals, and the decrease in absorbance at 320 nm was measured using a Nanodrop 1000 spectrophotometer (Thermo Fischer Scientific). For the kinetics study, the rate of MTX hydrolysis by purified proteins (2.12 µg/ml) was determined at different MTX concentrations (0.03 up to 0.42 mM) in 0.1 M Tris-HCl pH 7.3 and 0.2 mM ZnSO_4_ using Nunc 96 plates with flat bottom wells that were UV transparent. All reactions were carried out in triplicate at 37°C for 2 min and the decrease in absorbance at 320 nm was determined using Infinite M200 PRO NanoQuant Plate Reader (TECAN). Apparent K_m_, V_max_ and K_cat_ values of each protein were determined by fitting to the Michaelis-Menten equation using Graph pad PRISM 6 software. For serum stability assay, human serum was separated from normal blood samples and incubated with the purified fusion proteins (0.1 µl/µg) at 37°C for 14 days. Every 48 hours, a sample was taken and tested for CPG2 catalytic activity on MTX.

### Circular Dichroism spectroscopy of the generated fusion proteins

Circular Dichroism spectroscopy (CD) measurements of the single and double fusion proteins were performed using a Chirascan spectrometer (Applied Photophysics) for 10 µM protein (adjusted using the extension coefficient of each protein that were taken as ε = 23380 M^-1^ cm^-1^ for WT CPG2, 23505 M^-1^ cm^-1^ for single fusion protein and 23630 for double fusion protein measured using a Nanodrop 2000 spectrometer) in sterile water. The proteins were examined in a 0.2 mm SUPRASIL Quartz demountable rectangular (Hellma^®^) cuvette, with scanning between 180 and 260 nm at 25°C, band width of 0.5 nm, step size 1 nm and a scan time per point of 0.5 s. For each protein scan of repeat were averaged and smoothed using Chirascan™ analysis software. Water was used as a buffer baseline that was measured under the same parameters, then the final spectra of each protein were obtained by correcting them for the buffer baseline averaged spectra by subtraction.

In addition, secondary structure components of each protein were predicted for each corresponding measured spectrum by CD spectra deconvolution method using CDNN software (version 2.1) in the far UV spectral region in the 190–260 nm spectral region. For the deconvolution calculations, the number of amino acids of WT CPG2 was taken as 392 residues, and the molecular weight was 41,761.48 Da. For the single fusion protein, the number of amino acids was taken to be 403 residues and the molecular weight was 42,668.53 Da. For the double fusion protein, the number of amino acids was taken to be 410 and the molecular weight was 43,316.25 Da.

### *Ex-vivo* immunogenicity assay

The immunogenicity of the purified fusion proteins was determined using human peripheral blood mononuclear cells (PBMCs) and in conjunction with a proliferation assay. PBMCs from normal donors blood was separated and cultured in *X-VIVO*™ 15 media (1 × 10^6^ cells/ml in 96 well plate). 10 µl of a purified fusion protein was added to achieve a final concentration of 10 µg/ml. Prior to adding the fusion proteins, the level of endotoxin in the purified fusion protein samples was assessed using a Pierce LAL Chromogenic Endotoxin Quantitation Kit and, where necessary, high endotoxin levels were reduced using Pierce™ High Capacity Endotoxin Removal Resin to ˂ 0.1EU/ml. Negative controls were without any proteins added, while LPS was used as a positive control. PBMCs were incubated with the purified fusion proteins for 48 hrs at 37°C in a CO_2_ incubator. The T cell proliferation assay was performed using a CCK-8 kit, as indicated by the manufacturer. 10 µl of CCK-8 working solution was added to each well and the plate was incubated at 37°C for 4 hours. The resulting absorbance was then read at 450 nm using an Infinite M200 PRO NanoQuant Plate Reader (TECAN).

### Measurement of aminopeptidase N levels in various cancer cell lines

Cell lines were grown in 96 well plates using their recommended media and conditions (MDA-MB231, MCF-7, T47D, LoVo H661 A549, FaDu and SCC25 in RPMI-1640 supplemented with 10% FBS, HepG2, HCT and HT1080 in Minimum Essential Medium (MEM) supplemented with 10% FBS MDA-MB468 in Dulbecco’s Modified Eagle’s medium (DMEM) supplemented with 10% FBS). The cells were fixed with 4% paraformaldehyde for 15 minutes, and then blocked with 5% BSA in 1× PBS for one hour at room temperature. Next, the cells were incubated with mouse anti-CD13 antibody (WM15 ab7417), followed by washing twice with 1× PBS. The cells were then incubated with an anti-mouse antibody labelled with Alexa Fluor-488 and, before scanning, the cell nuclei were stained with DAPI. The cells were screened for the level of fluorescence using an Array Scan XTI instrument.

### Measurement of *in vitro* binding of fusion proteins to cancer cells differentially expressing APN

HT1080 and A549 cells were seeded at 25,000 cells/well density in 96 well plates and incubated at 37°C overnight. The next day the cells were fixed with 4% paraformaldehyde for 15 minutes at room temperature, followed by washing with 1 X PBST and blocking with 5% bovine serum albumin for 1 hour at room temperature. After washing with 1 X PBST three times, the cells were incubated with different concentrations of the purified fusion proteins (0.063, 0.127, 0.192 and 0.255 µM), at room temperature for 1 hourThis was followed by washing twice with 1 X PBST, addition of 100 µL (per well) anti-His6-HRP monoclonal antibody diluted (1:1000) in 1% BSA and incubation for 1 h at room temperature. After that the plate was washed with PBST twice, and the chromogenic HRP substrate TMB “3,3′,5,5′-Tetramethylbenzidine “ was added (100 µl/well). The reaction was stopped by adding 2N H_2_SO_4_, and the resulting color intensity was measured using an Infinite M200 PRO NanoQuant Plate Reader (TECAN) “OD at 450 nm”. GraphPad Prism was used to calculate the binding affinity and dissociation constants (Kd).

### Measurement of *in vitro* cytotoxicity of MTX and/or ZD2767P following treatment with fusion proteins

The cells were dispensed into 96 well plates at a density of 20,000 cells/well and allowed to attach overnight. The next day, the cells were incubated with 80 µg/ml of *Xen*. CPG2c proteins for two hours. The cells were washed at least twice with 1× PBS to remove any unbound proteins and treated with 5 nM of methotrexate (MTX) or different concentration of the prodrug ZD2767P (0.1, 0.5 and 1 µM). Several control groups were used for each cell line; one was treated only with the fusion proteins while others were treated only with MTX or with the prodrug.

The cells were grown and treated with fusion proteins for 2 hrs, washed and incubated with MTX or the prodrug ZD2767P for 1–2 hrs, after that washing and incubation of cells in complete media with no proteins or drugs for 48 hrs. 10 µl of 5 mg/ml MTT solution was added to the cells (final concentration 0.5 mg/ml) and incubated for 3–4 hrs. This was followed by washing cells with PBS and adding MTT solvent. After 15 min the absorbance of resulting color was measured at 570 nm.

### Data analysis

Data were processed using GraphPad Prism 5 software. Statistical analysis was carried with Student’s *t*-test, and results with *P* < 0.05 or *P* < 0.001 were defined as significant. One-way ANOVA with post hoc Tukey’s test were used to compare data of the APN expression level by cancer cell lines investigated, differences were considered significant when *P* < 0.001. For the binding affinity assay K_d_ was calculated using nonlinear regression analysis.

## CONCLUSIONS

Our work describes two CPG2 fusion proteins for the targeted treatment of aminopeptidase N expressing-cancer cells. Their characteristics suggest that it would be beneficial to further investigate their antitumor effects in animal models and possibly in clinical studies. Given the ease of production of such fusion proteins, their lower immunogenicity and the increased enzyme activity of the double fusion construct, they have the potential to be used in addition to, or replace, antibody-directed enzyme prodrug therapy. We name the modified strategy as ligand-directed enzyme prodrug therapy (LDEPT).
